# Actual Self-Image Versus Ideal Self-Image: An Exploratory Study of Self-Congruity Effects on Gambling Tourism

**DOI:** 10.3389/fpsyg.2021.588190

**Published:** 2021-07-08

**Authors:** Mao-Hua Li, Ivan Ka Wai Lai

**Affiliations:** ^1^Department of Economics and Management, Zhanjiang Preschool Education College, Zhanjiang, China; ^2^Faculty of International Tourism and Management, City University of Macau, Macau, China

**Keywords:** actual self-image, destination image, ideal self-image, self-congruity, gambling tourism, Macau

## Abstract

This study aims to apply self-congruity theory to examine the relationship between self-congruity of tourists and their perceived image of a gambling destination. This study employs the Euclidean distance model and extends Malhotra's pars of adjectives with five new items about gambling motives. A face-to-face questionnaire survey was used, and a total of 152 samples were collected from tourists in Macau. The results show that the actual self-image of tourists is more related to their perception of Macau image. For actual congruence, tourists exhibit a greater tendency to match the informal, liberal, and emotional image of Macau. For ideal congruence, they have a tendency to match the contemporary, organized, and pleasant image of Macau. This study makes up for the deficiency of self-congruity theory in tourism research. This study helps tourism departments to develop appropriate strategies to promote gambling tourism and disseminate relevant information that can bring gambling destinations closer to tourists.

## Introduction

Tourism, as a form of public leisure, has become the main source of economic income in many tourist destinations (Butowski, 2017). When competing with various tourist destinations, understanding how tourists select their destinations becomes a fundamental issue (Beerli et al., 2007). One of the important factors affecting the choice of tourist destinations is the image of the place (Baloglu and McCleary, 1999). Tourist destination image is the sum of tourists' cognitive and affective evaluations, which plays a positive role in destination marketing (San Martín and Del Bosque, 2008). However, for a gambling destination, say, Macau, its destination image is something special. Gambling in Macau officially began in 1847, Macau was called “Monte Carlo of the Orient” in the past (Kwan, 2004). Since 2006, Macau's gambling revenue has surpassed Las Vegas, and Macau became the top gambling destination in the world (Li et al., 2017). Macau is the former Portuguese colony, a small city about 32.9 square kilometers, and homes to 682.5 thousand people by the end of March 2021. For this East-Meets-West old city, Macau has two faces. On one side, there are a large number of modern casino resorts on Taipa Island. On the other side, there are 20 more sites listed as World Heritage by UNESCO on Macau semi-island. As other gambling cities, Macau government prevents using the words “casino” and “gambling” in promoting tourism. Most of the tourists returned from Macau are seldom talking about “casino” and “gambling.” Thus, ascertaining tourists' perceived image of Macau is crucial to the development and better adaptation to the policy changes (Li et al., 2017). To some extent, this can help the Macau government to better position gambling tourism and develop appropriate destination marketing strategies.

Previous studies in hospitality and tourism have used social psychology theories to understand tourist behavior. Self-congruity, as a core concept of social psychology, can help to explain the phenomenon involving tourism decision-making behavior (Pizam and Mansfeld, 1999). Self-congruity describes individual's psychological cognition and is widely applied in the field of consumer behavior and marketing to enable marketers to more accurately match customer needs and occupy market segments (Boksberger et al., 2011). In the context of tourism, the study of self-congruity of tourists can provide the destinations with strategic ideas of market positioning and advertising strategies (Sirgy and Su, 2000). According to self-congruity theory, tourists may prefer to visit a destination where it has a congruent image with self-image of tourists. In general, self-congruity of tourists influences their destination choice. But for Macau, due to its two-face image, how self-image of tourists associated with their perception of this destination image is still a question.

This study aims to investigate the association between self-image of tourists and their perceived image of a gambling destination—Macau. The contributions of this study are as follows: (1) It makes up for the deficiency of self-congruity theory in gambling tourism research; (2) it is helpful for gambling destinations to grasp their target tourist groups and implement targeted marketing strategies; and (3) it is helpful for gambling destinations to identify the focus of tourists' needs and for relevant tourism departments and enterprises to disseminate information that can bring the tourist destinations closer to the tourists.

## Literature Review

### Gambling Tourism

The term “tourism” has evolved throughout the Twentieth century (Panosso Netto et al., 2011). Today, the World Tourism Organization (UNWTO) defined tourism as a social, cultural, and economic phenomenon that entails the movement of people to countries or places outside their usual environment for personal or business/professional purposes (UNWTO, 2021). Therefore, the movement of people within their usual living spaces (e.g., work, school, shopping) and study and work trips are not considered tourism (Lohmann and Panosso Netto, 2017). As gambling can be a personal interest, then, for a person to go to another city for this interest, this movement can be regarded as a form of tourism.

There are three basic forms of tourism: domestic tourism, inbound tourism, and outbound tourism (UNWTO, 2021). Another common classification is based on travel motivations of tourists, such as leisure travel, business travel, and visiting friends and relatives (Chadwick, 1987; Shi et al., 2018). Among various forms of tourism, gambling tourism (or gaming tourism), like religious tourism, MICE tourism, and rural tourism, belongs to the category of alternative tourism. Gambling tourism entices large numbers of people to visit destinations with a high level of agglomeration of casinos and gaming sites (Metaxas and Folinas, 2016; Lai and Hitchcock, 2020). The gambling tourism industry is a comprehensive industry composed of travel agencies, hotels, casinos, entertainment venues, and other ancillary service industries, among which gambling plays a major role. Most tourists to gambling destinations are from the countries where gambling is illegal (Bonny-Noach and Sagiv-Alayoff, 2021). Hence, gambling tourism is a kind of tourism that tourists are attracted to a place by a combination of gambling and non-gambling activities (Lai and Hitchcock, 2020). Tourists participate in gambling, sightseeing, shopping, vacationing, and taking entertainment activities during a gambling trip (Rosenbaum and Wong, 2015). The motivations for them to go to a gambling destination are for increasing knowledge, exploring, sightseeing, shopping, taking entertainment, and participating in social activities (Lee et al., 2015; Ma and Lai, 2016). Therefore, players (gambles) who play casino games and use tourism-related services (transport, accommodation) are counted as (gaming) tourists. However, professional gamblers and pathological gamblers are not counted as tourists because they seek to carve out livelihoods from participation in gambling and do not join any non-gambling activities in the gambling destinations (Weinstock et al., 2013). For gambling destinations such as Las Vegas and Macau, casino gambling is respected as a form of tourism development because it can expand the destinations' dining, shopping, and entertainment attractiveness (Bowen, 2009).

### Gambling Destination

In 1931, Nevada legalized casinos state-wide, marking the beginning of the era of the casino in the world. In gambling cities, casino resorts were built as vacation venues to meet the diverse needs of tourists (Masiero et al., 2018). Casino resorts provide a great attraction to tourists because tourists can enjoy both hedonic and gambling activities there (Lai et al., 2020). Nowadays, with the fierce market competition, casino resorts become an effective marketing tool for attracting new tourists and increasing repeat customers for gambling destinations.

A number of studies discussed the development of gambling destinations in the United States, Macau, South Korea, Israel, and other countries. In the 1990's, Long (1995) studied the development of gambling in the United States and found that casino gambling makes contributions to the development of tourism. From a survey in the southern city of Eilat, Israeli and Mehrez (2000) found gambling as an important tourist attraction that could benefit Israel's economy. So they suggested that the policy-making department could make some attempts in the legalization of the casinos because it would bring a positive effect on economic development. For the development of casinos in Australia, Smeral (1998) argued that gambling provides tourists with diversified entertainment options and enriches their itinerary, thereby improving the structural competitiveness of a region. In Asia, Sheng and Tsui (2009) analyzed the relationship between Macau's gambling industry and urban development. They found that the rise and prosperity of the gambling industry are inseparable from the rational development strategy and the increasingly complete of the construction of the democratic system. Wan and Li (2013) indicated that in order to realize the sustainable development of Macau's tourism industry, it is necessary to develop from gambling to diversified development and formulate a comprehensive overall master plan. In conclusion, the development of gambling and tourism are complementary to each other, thereby attracting more tourists to gambling destinations. In addition, tourism development is an important driving force for the sustainable development of the gambling industry. Therefore, as far as gambling tourist destinations are concerned, general tourism and gambling tourism are inseparable, interdependent, and with mutual development.

### Destination Image and Gambling Destination Image

In the current dynamic and constantly changing market environment, the destination image is considered to be an important tool for enhancing the market competitiveness of tourist destinations (Afshardoost and Eshaghi, 2020). At present, the tourist destination image still lacks a unified concept, but most scholars study it from two directions. One is from the perspective of psychological activities of tourists. Hunt (1975) stated that destination image is the impression people hold on their non-residential place and is a purely subjective concept. Crompton (1979) believed that a destination image is the beliefs, ideas, and impressions formed by tourists on a destination. Baloglu and McCleary (1999) held that a tourist destination image refers to the individual's understanding, emotion, and impression of the tourist destination; it is a concept that expresses tourists' personal attitudes. Similarly, the conceptual model of tourist destination image proposed by Gallarza et al. (2002) also focused on the perspective of tourists. Moreover, Prayag and Ryan (2012) suggested that destination image conveys the individual's cognition and emotion, as well as behavioral feedback to the destination.

Another conceptual direction is from the perspective of the overall concept. Destination image could be viewed as a holistic aspect, which includes functional characteristics and psychological characteristics (Echtner and Ritchie, 1991, 1993). Therefore, destination image contains cognitive and affective elements that cover all the information related to a destination (Murphy et al., 2000), hence combining information sources, cultural factors, and social elements. Kislali et al. (2020) proposed an overall framework to explain the formation process of a destination image. No matter two (cognitive, affective) or three (cognitive, affective, and conative) components of destination image were defined by different scholars, they are apt to emphasize the nature of the holistic image of the tourist destination (Afshardoost and Eshaghi, 2020). Thus, the destination image reflects tourists' holistic evaluation of a destination (Josiassen et al., 2016; Afshardoost and Eshaghi, 2020). Furthermore, Kock et al. (2016) recognized the destination image as overall psychological evaluation of tourists toward the tourist destination. In view of the fact that most current studies in destination image were conducted from the perspective of psychological activities of tourists (e.g., Kock et al., 2016; Huete-Alcocer et al., 2019), thus, this research also studies the destination image from this perspective.

A gambling destination image can be regarded as adding the attributes of gambling on the basis of the general destination image. Since a gambling destination image is the destination characteristics perceived by tourists, so different gambling destinations have different gambling destination images (Masiero et al., 2018). Macau has a strong Portuguese colonial culture and a blend of Eastern and Western cultures. The casino resorts in Macau are gorgeous and luxurious. Its cuisine gives it a reputation of UNESCO's “Creative City of Gastronomy” (Yu and Sun, 2019). These characteristics not only play a differentiated role but also segment different gambling destinations. These characteristics are the source of tourists' self-perception of the gambling destination image.

### Self-Congruity

The term “self-concept” is considered to be the whole of the thoughts and feelings that the individual takes the self as the reference (Rosenberg, 1989). Self-congruity is an extension of self-concept, which plays a significant role in guiding and predicting people's attitudes and behaviors; it is widely applied in psychology, marketing, consumer behavior, and other fields (Sop and Kozak, 2019). Sirgy (1982) applied self-congruity to explain how self-concept and brand image (brand personality) interact with each other and influence the buying behavior of consumers. Self-congruity has been described as a matching process that the higher the degree of matching between the consumer's self-concept and the product image, the more likely it is to have a good impression of the product (the more likely it is to purchase the product) (Sirgy, 1985; Sirgy and Su, 2000; Sirgy et al., 2000). This point of view shows that in the current consumer market, individuals consume images rather than products themselves. This kind of behavior is consistent with their self-concept (Sop and Kozak, 2019). Therefore, consumers believe that their purchase behavior is self-expression, and they are inclined to choose things that reflect their images but refuse to choose things that do not conform to their images (Sirgy, 1985; Beerli et al., 2007; Branaghan and Hildebrand, 2011).

In terms of dimensionality, scholars initially classified the self-concept into actual self (how the individual actually perceives himself/herself to be) and ideal self (how the individual perceives himself/herself ideally be) (Dolich, 1969; Ross, 1971). Then, Sirgy (1982) introduced social self and ideal social self into the self-concept. The social self refers to how the individual thinks other people perceive himself/herself; however, the ideal social self represents how the individual wants others to perceive himself/herself. Bearden and Etzel (1982) believed that the formation and development of the social self were affected by the reference group. In order to gain the recognition of their relatives and friends, or to maintain all their current social relations, when consumers choose brands or products, they will strive to choose that they have the consistency between social self-concept and brand/product image (Escalas and Bettman, 2005). From the perspective of motivation, this reflects the social consistency need of social self-congruity and social recognition need of ideal social self-congruity (Sirgy, 2018). Therefore, researchers in brand marketing classified self-congruity into actual self-congruity, ideal self-congruity, social self-congruity, and ideal social self-congruity in their studies (Sirgy, 1985, 2018; Sirgy and Su, 2000; Luna-Cortés et al., 2019).

After Chon (1992) innovatively introduced self-congruity into tourism research, scholars began to study self-congruity on tourist behaviors. Sirgy and Su (2000) stated that self-congruity refers to the congruence between destination image and tourists' self-image. However, in tourism research, tourist behavior mostly reflects the motivation of self-consistency needs and self-esteem needs. Thus, researchers in tourism only tested actual self-congruity and ideal self-congruity associated with tourist destinations in their studies (e.g., Chon, 1992; Litvin and Goh, 2003; Beerli et al., 2007; Usakli and Baloglu, 2011; Kumar, 2016; Pan et al., 2017; Frías-Jamilena et al., 2019).

Previous studies in destination image found that self-congruity has an influence on travel behavior of tourists. The actual congruence (between destination image and actual self-image) and ideal congruence (between destination image and ideal self-image) directly affect satisfaction (Chon, 1992), experience (Hosany and Martin, 2012), word-of-mouth (Usakli and Baloglu, 2011), destination perceived value (Frías-Jamilena et al., 2019), and destination loyalty of tourists (Kumar, 2016). On the other hand, Kastenholz (2004) found that self-congruity had no significant influence on tourists' recommended intention, and Murphy et al. (2007) found no significant correlation between self-congruity and intention to revisit. The reasons for the inconsistent conclusions mainly include different research context, different self-congruity measurement methods, and whether the moderators are considered.

There are two measurement methods for measuring self-congruity: direct measurement and indirect measurement. The direct measurement method is based on tourists' overall and global perception of the tourist destination. Scholars initially used the direct method designed by Chon (1992), and later, Sirgy and Su (2000) further improved the method. By using a direct measurement method, Litvin and Goh (2002) found that self-congruity (actual and ideal) was significantly correlated with tourists' interest and the likelihood of visiting destinations. Usakli and Baloglu (2011) found that self-congruity (actual and ideal) had a significant effect on both revisiting and recommendation intentions. Recently, Frías-Jamilena et al. (2019) examined that self-congruity (actual and ideal) between tourists and destinations had a significant influence on the perceived value of destinations.

The indirect measurement method follows the calculation method of the absolute discrepancy index or Euclidean distance index. Sirgy and Danes (1982) pointed out that the two indices have no obvious difference. The semantic difference scale and Likert-type scale are two commonly used measurement tools to measure self-image and product/brand/destination image. Some of the measurement scales were tailored according to the research design (e.g., Sirgy, 1985; Ekinci and Riley, 2003; Ibrahim and Najjar, 2008), while other measurement scales used a standard set of image dimensions (e.g., Malhotra, 1981; Beerli et al., 2007; Hosany and Martin, 2012). Beerli et al. (2007) used a 7-point semantic differential scale, which was adapted from Malhotra (1981) and found that the more consistent the image of a destination is with the self-concept of tourists, the more likely the tourist is to visit the destination. Compared with the direct measurement, indirect measurement has its advantages. First, researchers can compare the differences between the consumer/tourist self-image and product/brand/destination image in each attribute, and then know which items are consistent between the two and which items are inconsistent. Second, the purpose of marketing is to define the product/brand/destination image as the most consistent image of consumers/tourists, and express and disseminate it, thereby strengthening the image while distinguishing it from the image of competitors. Therefore, it is necessary to select those attributes of the image that are most consistent with the self-concept of the target consumer/tourist and at the same time distinguish them from the image of competitors, and this method can help to discover those attributes. For a gambling destination, due to the particularity of gambling attributes, the image attributes for measuring the congruence between the gambling destination image and tourists' self-image should be different from those of other tourist destinations. Therefore, it is necessary to verify the relationship between the gambling destination image and self-image of tourists with an extended set of measurement attributes of self-congruity.

## Research Methods

### Research Questions

This study aims to extend our understanding of self-congruity applied in gambling destination image and empirically examines the relationship between tourists' self-image and the image of Macau. There are two key research questions: First, for the gambling destination of Macau, how consistency is tourists' actual self-image and ideal self-image (the differences between actual self-image and ideal self-image)? Second, how does Macau image relate to tourists' ideal self-image and actual self-image, respectively? Which one is more congruent?

### Scale Development

The scale to measure ideal self-image, actual self-image, and Macau image is adapted from Malhotra (1981), which consists of 15 bipolar adjectives as shown in Table 1. Since this study is related to a gambling destination, five measurable items are added for reflecting the image of the gambling destination. These adjectives are greedy-contented (Horch and Hodgins, 2013; Hing and Russell, 2017; Miller and Thomas, 2017), uncontrolled-controlled (Miller and Thomas, 2017; Nong et al., 2020), selfish-generous (Ohtsuka and Chan, 2009; Miller and Thomas, 2017; Brown and Russell, 2020), relaxed-stressed (Kneesel et al., 2010; Wong and Rosenbaum, 2012; Ma and Lai, 2016), and lucky-unlucky (Lee et al., 2006; Vong, 2007; Rodriguez et al., 2014). Since studies in gambling highlighted that players are greedy, uncontrolled, generous, stressed, and unlucky during gambling (Jefferson and Nicki, 2003; Ohtsuka and Chan, 2009; Ferentzy and Turner, 2013; Horch and Hodgins, 2013; Miller and Thomas, 2017; Brown and Russell, 2020), these five adjectives are included in this study. In total, the scale is composed of 20 bipolar adjectives.

**Table 1:** Scale items for measuring actual self-image, ideal self-image, and destination image.

**Scale item**	**References**
Rugged-delicate, excitable-calm, uncomfortable-comfortable, dominating-submissive, thrifty-indulgent, pleasant-unpleasant, contemporary-non-contemporary, organized-unorganized, rational-emotional, youthful-mature, formal-informal, orthodox-liberal, complex-simple, colorless-colorful, modest-vain	Malhotra, 1981
Greedy-contented	Horch and Hodgins, 2013; Miller and Thomas, 2017
Uncontrolled-controlled	Westphal, 2008; Miller and Thomas, 2017
Selfish-generous	Ohtsuka and Chan, 2009; Jones, 2013; Miller and Thomas, 2017
Relaxed-stressed	Stewart and Zack, 2008; Ma and Lai, 2016
Lucky-unlucky	Ferland et al., 2002; Rodriguez et al., 2014

For addressing the social desirability response bias, respondents are first asked to evaluate their ideal self-image by answering “Using the following list of adjectives, how would you ideally like to see yourself?” and then actual self-image by answering “How do you actually see yourself?.” Accordingly, respondents evaluated the destination image of Macau by answering “How do you think about Macau?” A 7-point semantic differential scale is used. Respondents make their choice according to the actual situation or real feelings and tick the appropriate number. Numbers “1” and “7” indicate that the adjectives at each end are very consistent with respondents' opinions. Number “4” indicates that they are not sure about this item. The closer the numbers are to each end, the more consistent their opinions are with the adjective.

The questionnaire is divided into three sections. In this study, the respondents are limited to tourists. The first section is a filter question: “Are you a tourist to Macau?” For professional gamblers and pathological gamblers, neither of them considers themselves tourists (Weinstock et al., 2013). As long as the respondents realize that they are tourists, this inclusion criterion can be met. The second section asked respondents to rate their perceptions of the ideal self-image, the image of Macau, and the actual self-image. The third section asked about the demographic information of the respondents. To ensure content validity, the questionnaire was examined by two university professors in this research field. Referring to their comments, the questionnaire was revised accordingly. The questionnaire was written in English and then translated into Chinese. In order to maximize the degree of translation equivalence, back-translation was used to identify any discrepancies in meaning or syntax (Mullen, 1995). Then, two doctoral students were invited to check the readability of the bilingual questionnaire. A pre-test was performed with 30 tourists in Macau in the first week of March 2019. The pre-test showed that the average response time for filling a questionnaire was <10 min, so the likelihood of non-response biases is minimized (Vercruyssen et al., 2011).

### Data Collection

Macau, one of the two Special Administrative Regions of the People's Republic of China, is located on the west side of the Pearl River Estuary, bordering Zhuhai City, Guangdong Province. It is positioning as a world tourism and leisure center. As a former Portuguese colony, the collision of Eastern and Western cultures makes Macau a unique city with a large number of historical and cultural relics. As a famous gambling destination, Macau is crowned for “the Las Vegas of the East,” where there are huge casinos packed with upscale resorts and high-end shopping malls. Also, its best cuisines, ancient architectures, and popular attractions attract a huge of tourists to visit. General tourism and gambling are the pillar industries. Therefore, Macau is an ideal site to be studied as a typical gambling tourist destination.

This study adopted a convenient sampling method to collect data from 10 a.m. to 7 p.m. in March 2019 in several places of Macau (the vicinities of border gates, attractions, and casino resorts). The interviewers first asked the respondents the filter questions: “Are you a tourist to Macau?” Only those who answered “yes” to the filter question were accepted to conduct the survey. Finally, 170 sets of questionnaires were obtained. However, 18 sets of questionnaires were invalid because respondents gave similar ratings to most items. In the end, a total of 152 sets of valid questionnaires were used for the final analysis.

### Analysis Method

This study used a generalized Euclidean distance model to obtain the scores of actual congruence (between actual self-image and image of Macau) and ideal congruence (between ideal self-image and image of Macau). Statistical analysis was performed with the SPSS version 24 package. A line chart was drawn with Microsoft Excel 2016. The Euclidean distance model was developed by Cronbach and Gleser (1953) and is generally used for studying self-congruity (e.g., Ross, 1971; Sirgy, 1982; Graeff, 1997; Abel et al., 2013). The congruence model is depicted as mathematically as follows:

$$\begin{array}{l} D=\sqrt{\overset{n}{\underset{i=1}{\sum }}{\left(P_{i}-S_{i}\right)}^{2}} \end{array}$$

In the above model, *i* is a particular image dimension. *P*_*i*_ and *S*_*i*_ are the perceptions of destination image and self-image on *i*. The degree of congruence (*D*) is the overall linear distance between self-image and destination image. If a tourist's self-image and the image of Macau are completely congruent, the *D*-value is zero. If the tourist's self-image and the image of Macau are more incongruent, the *D*-value will be high. This study follows Sirgy (1982) congruence model. His study only involved 168 female subjects. The sample size of this study is similar to Sirgy (1982). Since this study is an exploratory study, although the sample size is relatively small, it should be acceptable because the calculation of the degree of congruence is independent of the number of indicators.

## Results

### Respondent Characteristics

Table 2 summarizes the demographic profiles, of which 81 were males and 71 were females. The age group of 36–45 years (52, 34.3%) was the majority. Respondents were mainly accompanied by either their family or friends to Macau. As for the level of education, 61.8% of respondents had completed college or university education. Regarding the monthly income, the income for over half (55.9%) of respondents was over RMB 10,000 (USD 1= RMB 6.99). The majority of tourists visiting Macau were with vacation purpose; only a small portion of tourists was taking business trips or visiting relatives, etc.

**Table 2:** Description of respondents (*n* = 152).

		**Frequency**	**Percent**
Gender	Male	81	53.3
	Female	71	46.7
Age	18–25	18	11.8
	26–35	42	27.6
	36–45	52	34.3
	46–55	24	15.8
	55 or over	16	10.5
Education (completed)	High school and below	17	11.2
	Technical secondary school	21	13.8
	College/university	94	61.8
	Post-graduate	20	13.2
Monthly income (after tax) (RMB)	Less than 3,000	8	5.3
	3,001–6,000	17	11.2
	6,001–10,000	42	27.6
	10,001–15,000	45	29.6
	15,001–20,000	26	17.1
	20,001 or over	14	9.2
Main purpose	Business	12	7.9
	Visit relatives	10	6.5
	Vacation	124	81.7
	Other	6	3.9
Area	Mainland China	96	63.1
	Hong Kong	27	17.8
	Taiwan	13	8.6
	Overseas	16	10.5

### Self-Image Congruence and the Image of Macau

**Figure 1:**
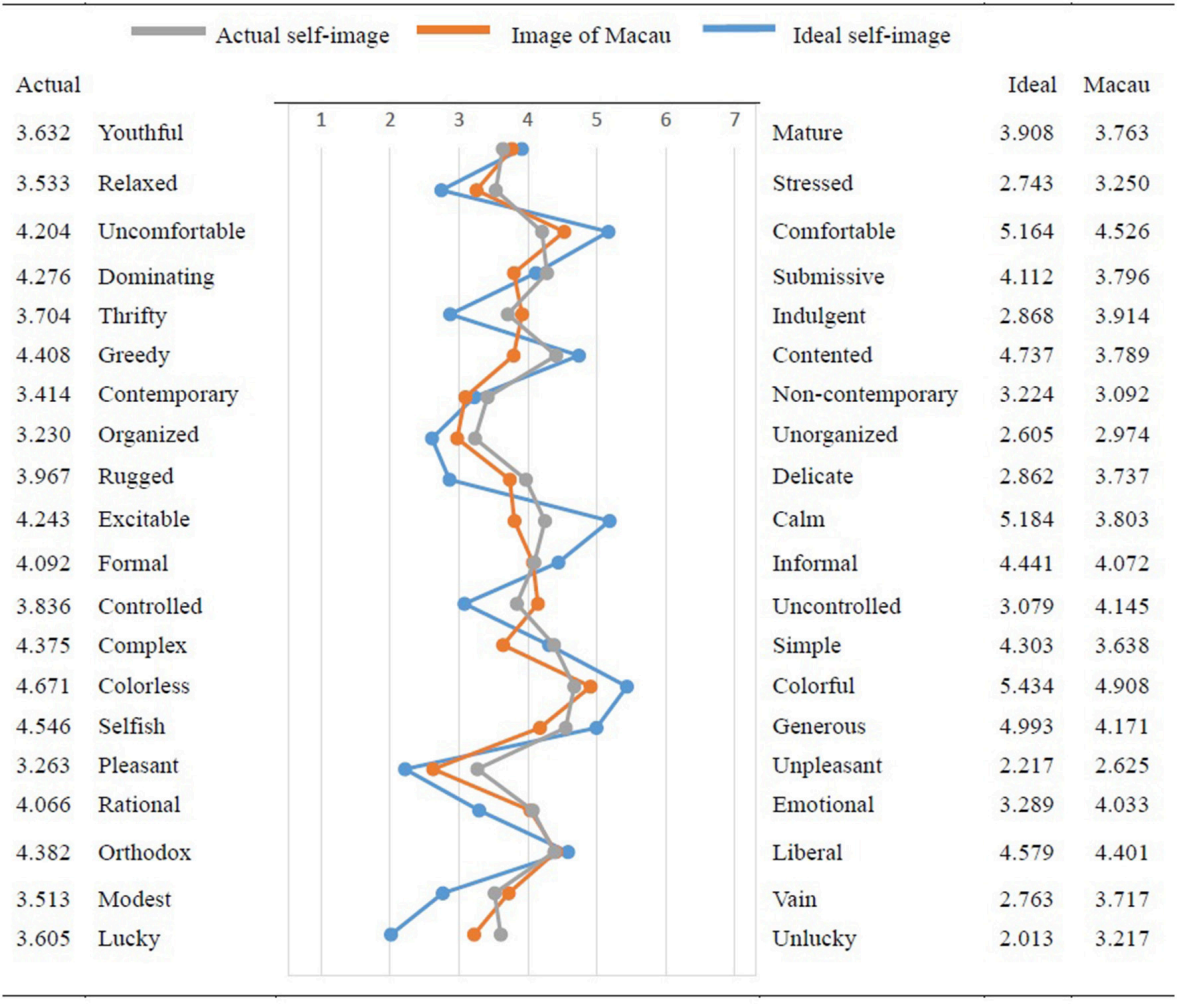
Actual self-image, image of Macau, and ideal self-image.

A scatter line chart is drawn to show the values of actual self-image, the image of Macau, and ideal self-image in 20 image dimensions as shown in Figure 1. Ten dimensions of the image of Macau are in-between the actual self-image and ideal self-image. The average, standard deviation, 1st quartile, median, and 3rd quartile of actual self-image are 3.948, 0.441, 3.551, 4.017, and 4.350 respectively. Participant's actual self-image tends to be organized (3.230), pleasant (3.263), generous (4.546), and colorful (4.671). The average, standard deviation, 1st quartile, median, and 3rd quartile of ideal self-image are 3.725, 1.080, 2.788, 3.599, and 4.698, respectively. Participant's ideal self-image tends to be lucky (2.013), pleasant (2.217), calm (5.184), and colorful (5.434).

The different values between compared profiles allow the description of a 20-item profile comparison in terms of the distance between two points. Paired samples *t*-test is performed to compare the tourists' actual self-image to their ideal self-image as shown in Table 3. “Diff” is the sample mean of the difference, and “S.D.” is the sample standard deviation of the difference. Only five image dimensions (complex, dominating, contemporary, orthodox, and youthful) show no difference (*p-*value > 0.05) between actual self-image and ideal self-image. The top three largest absolute differences are lucky (1.592), rugged (1.105), and pleasant (1.046).

**Table 3:** The consistency between actual self-image and ideal self-image.

		**Diff**.	**S.D**.	***t*-statistics**	***p*-value**
Complex	Simple	0.072	1.980	0.451	0.653
Dominating	Submissive	0.164	1.716	1.181	0.239
Contemporary	Non-contemporary	0.191	1.959	1.201	0.232
Orthodox	Liberal	−0.197	1.929	−1.261	0.209
Youthful	Mature	−0.276	2.719	−1.253	0.212
Greedy	Contented	−0.329	1.643	−2.469	0.015
Formal	Informal	−0.349	1.832	−2.347	0.020
Organized	Unorganized	0.625	1.956	3.940	0.000
Modest	Vain	0.750	1.524	6.069	0.000
Controlled	Uncontrolled	0.757	1.687	5.528	0.000
Colorless	Colorful	−0.763	1.413	−6.659	0.000
Rational	Emotional	0.776	1.920	4.986	0.000
Relaxed	Stressed	0.789	1.614	6.031	0.000
Selfish	Generous	−0.822	1.856	−5.462	0.000
Thrifty	Indulgent	0.836	1.580	6.520	0.000
Excitable	Calm	−0.941	1.739	−6.671	0.000
Uncomfortable	Comfortable	−0.961	1.852	−6.395	0.000
Pleasant	Unpleasant	1.046	1.766	7.305	0.000
Rugged	Delicate	1.105	1.798	7.577	0.000
Lucky	Unlucky	1.592	1.567	12.528	0.000

Congruence or lack of congruence among the profiles can be represented by the magnitude of the computed absolute difference value as shown in Table 4. For examining the actual self-image congruence with the image of Macau (referred to as actual congruence), the top three smallest absolute values between tourists' actual self-image and the image of Macau are formal (0.020), liberal (0.020), and emotional (0.033). For measuring the ideal self-image congruence with the image of Macau (referred to as ideal congruence), the top three smallest absolute values between tourists' ideal self-image and the image of Macau are contemporary (0.132), youthful (0.145), and liberal (0.178). Table 4 shows that there are 16 items whose actual congruence is smaller than ideal congruence. By applying the Euclidean distance model, the degree of actual congruence is 1.647. The degree of ideal congruence is 3.373. The degree of actual congruence is smaller than the degree of ideal congruence.

**Table 4:** Profile of ideal congruence and actual congruence.

		**Ideal congruence**		**Actual congruence**	
Youthful	Mature		0.145		0.132
Relaxed	Stressed		0.507		0.283
Uncomfortable	Comfortable		0.638		0.322
Dominating	Submissive	0.316		0.480	
Thrifty	Indulgent		1.046		0.211
Greedy	Contented		0.947		0.618
Contemporary	Non-contemporary	0.132		0.322	
Organized	Unorganized		0.368		0.257
Rugged	Delicate		0.875		0.230
Excitable	Calm		1.382		0.441
Formal	Informal		0.368		0.020
Controlled	Uncontrolled		1.066		0.309
Complex	Simple	0.664		0.737	
Colorless	Colorful		0.526		0.237
Selfish	Generous		0.822		0.375
Pleasant	Unpleasant	0.408		0.638	
Rational	Emotional		0.743		0.033
Orthodox	Liberal		0.178		0.020
Modest	Vain		0.954		0.204
Lucky	Unlucky		1.204		0.388

## Discussion and Conclusion

### Conclusion

For the consistency of tourists' actual self-image and ideal self-image, 15 out of 20 bipolar items have a significant difference. The largest different image dimensions are related to gambling such as lucky, rugged, and pleasant. From Figure 1, the other five image dimensions show little difference between actual self-image and ideal self-image and also little difference with the image of Macau.

Sixteen out of 20 bipolar items of tourists' actual self-image match with the image of Macau more than tourists' ideal self-image. These 16 items include five added items, which are related to a gambling destination. Only the image dimensions about Macau's current city development such as contemporary and organized match tourists' ideal self-image. The degree of actual congruence is smaller than the degree of ideal congruence.

### Theoretical Implications

This study successfully applies the self-congruity theory in gambling tourism research. It contributes to our knowledge in understanding the relationship between tourists' self-image and gambling destination image. According to this research results, the consistency of self-image in the actual dimension between tourists and Macau is higher than that in the ideal dimension. It implies that actual self-congruence takes an important role in promoting Macau's destination image. These findings are inconsistent with Hosany (2010) study for which ideal self-congruence shows a stronger effect on cruisers' experience than the actual self-congruence. However, in Huang et al. (2017) recent study in Yangshuo, China, tourists' ideal self conforms more to the actual self during the destination brand attachment development process. It seems that the effects of ideal self-image and actual self-image are different in different forms of tourism. This study is the pioneer of using self-congruity theory in the field of gambling tourism research, so follow-up researchers can conduct further research based on this research setting.

The study contributes to the extension of Malhotra (1981) scale by adding five added image attributes related to gambling. The values rated for the ideal self-image of these five items are far from the central point (4.0). Tourists are ideally seeking lucky (2.013), generous (4.993), contented (4.737), relaxed (2.743), and controlled (3.079) in Macau. For relieving tension, tourists go to travel. Tourists in Macau feel relaxed and contented. When tourists are visiting casinos, they psychologically hope to be lucky enough to win in gambling (Rodriguez et al., 2014). During gambling, they show generously because they psychologically prepare to “win some, lose some.” However, they need to have self-control for preventing unaffordable lost. Tourists (gamblers) with harmonious passion can control their engagement in gambling activities (Back et al., 2011). With self-control, gambling is a recreational activity. Without self-control, gambling is a pathological activity. These five image attributes present a harmony between passion and control when tourists are playing casino games. Therefore, for studying self-congruity effects on gambling tourism, these five attributes are essential to present the image of tourists and gambling destinations. The extended image attributes are useful for researchers applied in future studies in gambling destinations.

As mentioned in the literature, researchers such as Huang et al. (2017) commonly employed a direct measurement method to compare the effects of ideal self-congruence and actual self-congruence on tourists' attitudes and behaviors. This study applies the indirect measurement method to measure the Euclidean distance index. This method can find the most consistent attributes with the self-concept of the target tourists. This study reveals the association between tourists' self-image and the gambling destination image. For the whole 20 bipolar items, 16 of them go to actual congruence. Of 16 image attributes, Macau images of liberal (0.020), formal/informal (0.020), and emotional (0.033) are more actual congruent. Liberal can be seen from many aspects of Macau because Macau is a liberal society (Sheng, 2016). Referring to the Macau tourism industry development master plan, Macau is formally positioning as the World Center of Tourism and Leisure (Liu et al., 2021). It is working hard to cleanse the gambling image leftover from history. Tourists in Macau can have rich emotional experiences, including joy, positive surprise, love, caring, negative surprise, and lost (Lai et al., 2020). Thus, these image attributes strongly connect with tourists' mind and influence tourists psychologically bonded with Macau. On the other hand, tourists in Macau perceived a high ideal congruence in contemporary (0.132). They enjoy their vacations at the Cotai Strip where it is laid on the reclaimed land. Many luxurious resorts and various man-made wonders stand in there. Tourists yearn for a better life and pursue a better experience at luxurious resorts in Macau. Therefore, the contemporary image of Macau accords with tourists' pursuit and yearning for the ideal self. This study successfully identifies the consistent image attributes that can match tourists' self-concept to a destination image. Knowing these consistent image attributes is more useful for tourism market development practices.

### Practical Implications

Through image marketing, destination marketers can firmly grasp the hearts of tourists, thereby promoting the development of tourism. However, the issue is what the attributes of tourists' self-image can be linked to their perception of the destination image. By finding the consistent image attribute with the self-concept, this study helps the Macau government to better position the gambling tourism and develop appropriate destination marketing strategies. From Figure 1, three image dimensions are almost overlapped. It means that tourists' actual and ideal self-images match with the image of Macau in liberal, contemporary, and youthful/mature. According to self-congruity theory, tourists tend to visit a place where its image has a greater congruency between their self-image (Beerli et al., 2007). The Macau government tourism office should start from the tourists' self-concept to formulate its marketing strategies in highlighting Macau as a city where it is liberal, contemporary, and a balance between youthful and mature. Then, the tourism office can use social media to awaken the expression of tourists' actual self-concept, meet the consistency of the target tourists' actual self-concept, and enhance the attraction of tourist destinations, thereby inspiring tourists' travel behaviors. For example, the tourism office can develop a series of Tiktok videos in the theme of each image attribute.

For the casino resort operators, the issue is high competition in similar services. This study helps them determine the focus of customer needs and implement targeted marketing strategies to delight customers. Since the image of Macau is closer to the actual self-image of tourists, from the casino resort marketing perspective, marketers should emphasize those image dimensions that are highly actual congruence when making promotions of casino resorts. When doing advertisements, images of comfortable, contended, colorful, liberal, and delicate can be used to meet tourists' actual needs. The current advertisements are too focused on promoting the ideal self-image (contemporary—existing facilities and services) of the casino resorts. It gives people the feeling of being too philistine and lacking diversified elements. From the marketing campaigns of the casino resorts, the casino resort operators should combine the unique characteristics of the casino resorts to grasp the actual self-concept expression of the target tourists. By creating gambling products to meet the self-congruence of the target tourists, the casino resorts can maintain a stable target tourist group. For example, casino resorts can be configured a micro-film with a storyline describing the exciting experiences with consistent self-image elements in a journey at the casino resorts in Macau.

### Limitations and Further Research Directions

First, this study only investigates tourists' perception of self-congruity on gambling destination image; future studies should incorporate satisfaction and intention to recommend in modeling experiences of tourists. Second, previous research establishes the two common methods to measure self-congruity: the indirect method and the direct method. This study uses an indirect measurement method. The direct measurement method also has some advantages in the overall operation, which can directly measure the degree of congruence through the questions instead of using bipolar adjectives (Sirgy et al., 1997; Hosany and Martin, 2012). Subsequent studies may attempt to use direct measurement for comparison. Third, this study uses a one-time questionnaire survey of tourists in Macau; further studies could collect data from tourists at different times of the year. Moreover, different gambling destinations have different characteristics, so the image congruence of tourists' perception will be different. Therefore, further studies should be conducted in other gambling tourist destinations. The use of convenience samples is a limitation of the study. Probability sampling is recommended further. Finally, the sample size of this exploratory study is small; further studies are recommended with a large sample size to compare any differences between gaming tourists and non-gaming tourists.

## Data Availability Statement

The raw data supporting the conclusions of this article will be made available by the authors, without undue reservation.

## Ethics Statement

Ethical review and approval was not required for the study on human participants in accordance with the local legislation and institutional requirements. Written informed consent for participation was not required for this study in accordance with the national legislation and the institutional requirements.

## Author Contributions

M-HL and IL jointly contributed to the development of the research framework. M-HL collected the data and drafted the manuscript. IL directed the paper writing and revised the manuscript. All authors contributed to the article and approved the submitted version.

## Conflict of Interest

The authors declare that the research was conducted in the absence of any commercial or financial relationships that could be construed as a potential conflict of interest.

## References

[B1] Abel J. I. Buff C. L. O'Neill J. C. (2013). Actual self-concept versus ideal self-concept. Sport Bus. Manag. 3, 78–96. 10.1108/20426781311316915

[B2] Afshardoost M. Eshaghi M. S. (2020). Destination image and tourist behavioural intentions: a meta-analysis. Tourism Manag. 81:104154. 10.1016/j.tourman.2020.104154

[B3] Back K. J. Lee C. K. Stinchfield R. (2011). Gambling motivation and passion: a comparison study of recreational and pathological gamblers. J. Gambl. Stud. 27, 355–370. 10.1007/s10899-010-9212-2 20680417

[B4] Baloglu S. McCleary K. W. (1999). A model of destination image formation. Ann. Tourism Res. 26, 868–897. 10.1016/S0160-7383(99)00030-4

[B5] Bearden W. O. Etzel M. J. (1982). Reference group influence on product and brand purchase decisions. J. Consum. Res. 9, 183–194. 10.1086/208911

[B6] Beerli A. Meneses G. D. Gil S. M. (2007). Self-congruity and destination choice. Ann. Tourism Res. 34, 571–587. 10.1016/j.annals.2007.01.005

[B7] Boksberger P. Dolnicar S. Laesser C. Randle M. (2011). Self-congruity theory: to what extent does it hold in tourism? J. Travel Res. 50, 454–464. 10.1177/0047287510368164

[B8] Bonny-Noach H. Sagiv-Alayoff M. (2021). Casino tourism destinations: health risk for travellers with gambling disorder and related medical conditions. J. Travel Med. 28:taaa147. 10.1093/jtm/taaa147 32856061

[B9] Bowen J. (2009). Casinos as an antecedent of tourism development. Worldwide Hosp. Tourism Themes 1, 332–343. 10.1108/17554210911002174

[B10] Branaghan R. J. Hildebrand E. A. (2011). Brand personality, self-congruity, and preference: a knowledge structures approach. J. Consum. Behav. 10, 304–312. 10.1002/cb.365

[B11] Brown K. L. Russell A. M. (2020). What can be done to reduce the public stigma of gambling disorder? Lessons from other stigmatised conditions. J. Gambl. Stud. 36, 23–38. 10.1007/s10899-019-09890-9 31520273

[B12] Butowski L. (2017). Tourist sustainability of destination as a measure of its development. Curr. Issues Tourism 22, 1043–1061. 10.1080/13683500.2017.1351926

[B13] Chadwick R. (1987). Concepts, definitions, and measures used in travel and tourism research, in Travel, Tourism and Hospitality Research: A Handbook for Managers and Hospitality Researchers, eds Brent Richie J. R. Goeldner C. (New York, NY: Wiley), 101–116.

[B14] Chon K. S. (1992). Self-image/destination image congruity. Ann. Tourism Res. 19, 360–363. 10.1016/0160-7383(92)90090-C

[B15] Crompton J. L. (1979). An assessment of the image of Mexico as a vacation destination and the influence of geographical location upon that image. J. Travel Res. 17, 18–23. 10.1177/004728757901700404

[B16] Cronbach L. J. Gleser G. C. (1953). Assessing similarity between profiles. Psychol. Bull. 50, 456–473. 10.1037/h0057173 13112334

[B17] Dolich I. J. (1969). Congruence relationships between self images and product brands. J. Mark. Res. 6, 80–84. 10.1177/002224376900600109

[B18] Echtner C. M. Ritchie J. R. B. (1991). The meaning and measurement of destination image. J. Tourism Stud. 2, 2–12.

[B19] Echtner C. M. Ritchie J. R. B. (1993). The measurement of destination image: an empirical assessment. J. Travel Res. 31, 3–13. 10.1177/004728759303100402

[B20] Ekinci Y. Riley M. (2003). An investigation of self-concept: actual and ideal self-congruence compared in the context of service evaluation. J. Retail. Consum. Serv. 10, 201–214. 10.1016/S0969-6989(02)00008-5

[B21] Escalas J. E. Bettman J. R. (2005). Self-construal, reference groups, and brand meaning. J. Consum. Res. 32, 378–389. 10.1086/497549

[B22] Ferentzy P. Turner N. (2013). The History of Problem Gambling. New York, NY: Springer.

[B23] Ferland F. Ladouceur R. Vitaro F. (2002). Prevention of problem gambling. J. Gambl. Stud. 18, 19–29. 10.1023/A:1014528128578 12050845

[B24] Frías-Jamilena D. M. Castañeda-García J. A. Barrio-García S. D. (2019). Self-congruity and motivations as antecedents of destination perceived value: the moderating effect of previous experience. Int. J. Tourism Res. 21, 23–36. 10.1002/jtr.2238

[B25] Gallarza M. G. Saura I. G. Garcia H. C. (2002). Destination image: towards a conceptual framework. Ann. Tourism Res. 29, 56–78. 10.1016/S0160-7383(01)00031-7

[B26] Graeff T. R. (1997). Consumption situations and the effects of brand image on consumers' brand evaluations. Psychol. Mark. 14, 49–70. 10.1002/(SICI)1520-6793(199701)14:1<49::AID-MAR4>3.0.CO;2-O

[B27] Hing N. Russell A. M. (2017). How anticipated and experienced stigma can contribute to self-stigma: the case of problem gambling. Front. Psychol. 8:235. 10.3389/fpsyg.2017.00235 28270787 PMC5318456

[B28] Horch J. Hodgins D. (2013). Stereotypes of problem gambling. J. Gambl. Issues 28, 1–19. 10.4309/jgi.2013.28.10

[B29] Hosany S. (2010). The effects of self-image congruence, tourists' experiences and satisfaction on behavioral intention, in The Proceedings of 2010 TTRA International Conference (Wheat Ridge, CO: Travel and Tourism Research Association: Advancing Tourism Research Globally). Available online at: https://scholarworks.umass.edu/ttra/2010/Oral/4 (accessed May 29, 2021).

[B30] Hosany S. Martin D. (2012). Self-image congruence in consumer behavior. J. Bus. Res. 65, 685–691. 10.1016/j.jbusres.2011.03.015

[B31] Huang Z. Zhang C. Hu J. (2017). Destination brand personality and destination brand attachment – the involvement of self-congruence. J. Travel Tourism Mark. 34, 1198–1210. 10.1080/10548408.2017.1330171

[B32] Huete-Alcocer N. Martinez-Ruiz M. P. López-Ruiz V. R. Izquiedo-Yusta A. (2019). Archeological tourist destination image formation: influence of information sources on the cognitive, affective and unique image. Front. Psychol. 10:2382. 10.3389/fpsyg.2019.02382 31695658 PMC6817941

[B33] Hunt J. D. (1975). Image as a factor in tourism development. J. Travel Res. 13, 1–7. 10.1177/004728757501300301

[B34] Ibrahim H. Najjar F. (2008). Assessing the effects of self-congruity, attitudes and customer satisfaction on customer behavioural intentions in retail environment. Mark. Intell. Plann. 26, 207–227. 10.1108/02634500810860638

[B35] Israeli A. A. Mehrez A. (2000). From illegal gambling to legal gaming: casinos in Israel. Tourism Manag. 21, 281–291. 10.1016/S0261-5177(99)00059-X

[B36] Jefferson S. Nicki R. (2003). A new instrument to measure cognitive distortions in video lottery terminal users: the Informational Biases Scale (IBS). J. Gambl. Stud. 19, 387–403. 10.1023/A:1026327926024 14634299

[B37] Jones D. N. (2013). What's mine is mine and what's yours is mine: the dark triad and gambling with your neighbor's money. J. Res. Pers. 47, 563–571. 10.1016/j.jrp.2013.04.005

[B38] Josiassen A. Assaf A. G. Woo L. Kock F. (2016). The imagery–image duality model: an integrative review and advocating for improved delimitation of concepts. J. Travel Res. 55, 789–803. 10.1177/0047287515583358

[B39] Kastenholz E. (2004). Assessment and role of destination-self-congruity. Ann. Tourism Res. 31, 719–723. 10.1016/j.annals.2003.11.003

[B40] Kislali H. Kavaratzis M. Saren M. (2020). Destination image formation: towards a holistic approach. Int. J. Tourism Res. 22, 266–276. 10.1002/jtr.2335

[B41] Kneesel E. Baloglu S. Millar M. (2010). Gaming destination images: implications for branding. J. Travel Res. 49, 68–78. 10.1177/0047287509336474

[B42] Kock F. Josiassen A. Assaf A. G. (2016). Advancing destination image: the destination content model. Ann. Tourism Res. 61, 28–44. 10.1016/j.annals.2016.07.003

[B43] Kumar V. (2016). Examining the role of destination personality and self-congruity in predicting tourist behavior. Tourism Manag. Perspect. 20, 217–227. 10.1016/j.tmp.2016.09.006

[B44] Kwan F. V. C. (2004). Gambling attitudes and gambling behavior of residents of Macao: the monte carlo of the orient. J. Travel Res. 42, 271–278. 10.1177/0047287503254956

[B45] Lai I. K. W. Hitchcock M. (2020). How gaming tourism affects tourism development through word-of-mouth communication regarding a destination: applying the integrated satisfaction theory. Asia Pac. J. Tourism Res. 25, 610–626. 10.1080/10941665.2020.1752748

[B46] Lai I. K. W. Yang T. Hitchcock M. (2020). Evaluating tourists' emotional experiences regarding destination casino resorts: an impact-asymmetry analysis. J. Destination Mark. Manag. 16:100365. 10.1016/j.jdmm.2019.05.008

[B47] Lee C.-K. Lee Y.-K. Bernhard B. J. Yoon Y.-S. (2006). Segmenting casino gamblers by motivation: a cluster analysis of Korean gamblers. Tourism Manag. 27, 856–866. 10.1016/j.tourman.2005.05.00931325014

[B48] Lee C. K. Bernhard B. J. Kim J. Fong T. Lee T. K. (2015). Differential gambling motivations and recreational activity preferences among casino gamblers. J. Gambl. Stud. 31, 1833–1847. 10.1007/s10899-014-9513-y 25398482

[B49] Li J. Kim W. G. Wong I. A. (2017). Does destination perception differ based on traveler type? A case of the world gambling capital: Macau. Tourism Plann. Dev. 14, 15–30. 10.1080/21568316.2016.1152289

[B50] Litvin S. W. Goh H. (2002). Self-image congruity: a valid tourism theory? Tourism Manag. 23, 81–83. 10.1016/S0261-5177(01)00065-6

[B51] Litvin S. W. Goh H. K. (2003). Individualism/collectivism as a moderating factor to the self-image congruity concept. J. Vacat. Mark. 10, 23–32. 10.1177/135676670301000103

[B52] Liu M. T. Liu Y. Mo Z. Ng K. L. (2021). Using text mining to track changes in travel destination image: the case of Macau. Asia Pac. J. Mark. Logist. 33, 371–393. 10.1108/APJML-08-2019-0477

[B53] Lohmann G. Panosso Netto A. (2017). Tourism Theory: Concepts, Models, and Systems. Oxfordshire: CABI. 10.1079/9781780647159.0000

[B54] Long P. T. (1995). Casino gaming in the United States: 1994 status and implications. Tourism Manag. 16, 189–197. 10.1016/0261-5177(95)00013-E

[B55] Luna-Cortés G. López-Bonilla J. M. López-Bonilla L. M. (2019). Self-congruity, social value, and the use of virtual social networks by generation y travelers. J. Travel Res. 58, 398–410. 10.1177/0047287518755502

[B56] Ma E. Lai I. K. W. (2016). Gambling motivation among tourists in Macau's casino resorts. Asia Pac. J. Tourism Res. 21, 1227–1240. 10.1080/10941665.2016.1140661

[B57] Malhotra N. K. (1981). A scale to measure self-concepts, person concepts, and product concepts. J. Mark. Res. 18, 456–464. 10.1177/002224378101800407

[B58] Masiero L. Qian J. Fong D. Law R. (2018). Gambling destinations and the effect of gambling results on tourist satisfaction and loyalty. J. Travel Tourism Mark. 35, 678–689. 10.1080/10548408.2017.1403411

[B59] Metaxas T. Folinas S. (2016). Gambling Tourism and Economic Development: Some Lessons From Macao. MPRA Paper No. 72397. University Library of Munich.

[B60] Miller H. E. Thomas S. (2017). The “walk of shame”: a qualitative study of the influences of negative stereotyping of problem gambling on gambling attitudes and behaviours. Int. J. Ment. Health Addict. 15, 1284–1300. 10.1007/s11469-017-9749-8

[B61] Mullen M. R. (1995). Diagnosing measurement equivalence in cross-national research. J. Int. Bus. Stud. 26, 573–596. 10.1057/palgrave.jibs.8490187

[B62] Murphy L. Benckendorff P. Moscardo G. (2007). Linking travel motivation, tourist self-image and destination brand personality. J. Travel Tourism Mark. 22, 45–59. 10.1300/J073v22n02_04

[B63] Murphy P. Pritchard M. P. Smith B. (2000). The destination product and its impact on traveller perceptions. Tourism Manag. 21, 43–52. 10.1016/S0261-5177(99)00080-1

[B64] Nong S. Z. Fong L. H. N. Fong D. K. C. Lam D. (2020). Segmenting Chinese gamblers based on gambling forms: a latent class analysis. J. Gambl. Stud. 36, 141–159. 10.1007/s10899-019-09877-6 31325014

[B65] Ohtsuka K. Chan C. C. (2009). Desperate housewives: an analysis of the characterisations of female gamblers portrayed in gambling movies in Hong Kong. Int. J. Ment. Health Addict. 7, 229–238. 10.1007/s11469-008-9180-2

[B66] Pan L. Zhang M. Gursoy D. Lu L. (2017). Development and validation of a destination personality scale for mainland Chinese travelers. Tourism Manag. 59, 338–348. 10.1016/j.tourman.2016.08.005

[B67] Panosso Netto A. Tomillo Noguero F. Jager M. (2011). Por uma visão crítica nos estudos turísticos. Turismo Análise 22, 539–560. 10.11606/issn.1984-4867.v22i3p539-560

[B68] Pizam A. Mansfeld Y. (1999). Consumer Behavior in Travel and Tourism. Binghamton, NY: Hawthorn Hospitality Press.

[B69] Prayag G. Ryan C. (2012). Antecedents of tourists' loyalty to mauritius. J. Travel Res. 51, 342–356. 10.1177/0047287511410321

[B70] Rodriguez L. M. Neighbors C. Rinker D. V. Tackett J. L. (2014). Motivational profiles of gambling behavior: self-determination theory, gambling motives, and gambling behavior. J. Gambl. Stud. 31, 1597–1615. 10.1007/s10899-014-9497-7 25129824

[B71] Rosenbaum M. S. Wong I. A. (2015). When gambling is healthy: the restorative potential of casinos. J. Serv. Mark. 29, 622–633. 10.1108/JSM-01-2015-0025

[B72] Rosenberg M. (1989). Self-concept research: a historical overview. Soc. Forces 68, 34–44. 10.2307/2579218

[B73] Ross I. (1971). Self-concept and brand preference. J. Bus. 44, 38–50. 10.1086/295331

[B74] San Martín H. Del Bosque I. A. R. (2008). Exploring the cognitive-affective nature of destination image and the role of psychological factors in its formation. Tourism Manag. 29, 263–277. 10.1016/j.tourman.2007.03.012

[B75] Sheng L. (2016). The transformation of island city politics: the case of Macau. Island Stud. J. 11, 521–536. Available online at: https://www.islandstudies.ca/sites/islandstudies.ca/files/ISJ-11-2-MS362-LiSheng.pdf

[B76] Sheng L. Tsui Y. (2009). Casino boom and local politics: the city of Macao. Cities 26, 67–73. 10.1016/j.cities.2009.01.002

[B77] Shi T. Liu C. Li J. (2018). Market segmentation by travel motivations under a transforming economy: evidence from the Monte Carlo of the orient. Sustainability 10:3395. 10.3390/su10103395

[B78] Sirgy M. J. (1982). Self-concept in consumer behavior: a critical review. J. Consum. Res. 9, 287–300. 10.1086/208924

[B79] Sirgy M. J. (1985). Using self-congruity and ideal congruity to predict purchase intention. J. Bus. Res. 13, 195–206. 10.1016/0148-2963(85)90026-8

[B80] Sirgy M. J. (2018). Self-congruity theory in consumer behavior: a little history. J. Glob. Scholars Mark. Sci. 28, 197–207. 10.1080/21639159.2018.1436981

[B81] Sirgy M. J. Danes J. E. (1982). Self-Image/Product-Image Congruence Models: Testing Selected Models. ACR North American Advances.

[B82] Sirgy M. J. Grewal D. Mangleburg T. (2000). Retail environment, self-congruity, and retail patronage. J. Bus. Res. 49, 127–138. 10.1016/S0148-2963(99)00009-0

[B83] Sirgy M. J. Grewal D. Mangleburg T. F. Park J.-O. Chon K.-S. Claiborne C. B.. (1997). Assessing the predictive validity of two methods of measuring self-image congruence. J. Acad. Mark. Sci. 25, 229–241. 10.1177/0092070397253004

[B84] Sirgy M. J. Su C. (2000). Destination image, self-congruity, and travel behavior: toward an integrative model. J. Travel Res. 38, 340–352. 10.1177/004728750003800402

[B85] Smeral E. (1998). Economic aspects of casino gaming in Austria. J. Travel Res. 36, 33–39. 10.1177/004728759803600404

[B86] Sop S. A. Kozak N. (2019). Effects of brand personality, self-congruity and functional congruity on hotel brand loyalty. J. Hospitality Mark. Manag. 28, 926–956. 10.1080/19368623.2019.1577202

[B87] Stewart S. H. Zack M. (2008). Development and psychometric evaluation of a three-dimensional gambling motives questionnaire. Addiction 103, 1110–1117. 10.1111/j.1360-0443.2008.02235.x 18554344

[B88] UNWTO (2021). Glossary of tourism terms. UNWTO. Available online at: https://www.unwto.org/glossary-tourism-terms (accessed May 29, 2021).

[B89] Usakli A. Baloglu S. (2011). Brand personality of tourist destinations: an application of self-congruity theory. Tourism Manag. 32, 114–127. 10.1016/j.tourman.2010.06.006

[B90] Vercruyssen A. Roose H. Van de Putte B. (2011). Underestimating busyness: indications of nonresponse bias due to work–family conflict and time pressure. Soc. Sci. Res. 40, 1691–1701. 10.1016/j.ssresearch.2011.06.004

[B91] Vong F. (2007). The psychology of risk-taking in gambling among Chinese visitors to Macau. Int. Gambl. Stud. 7, 29–42. 10.1080/14459790601157731

[B92] Wan Y. K. P. Li X. (2013). Sustainability of tourism development in Macao, China. Int. J. Tourism Res. 15, 52–65. 10.1002/jtr.873

[B93] Weinstock J. Massura C. E. Petry N. M. (2013). Professional and pathological gamblers: similarities and differences. J. Gambl. Stud. 29, 205–216. 10.1007/s10899-012-9308-y 22581197

[B94] Westphal J. R. (2008). Pathological gambling: psychiatric models. Int. J. Ment. Health Addict. 6, 602–618. 10.1007/s11469-008-9166-0

[B95] Wong I. A. Rosenbaum M. S. (2012). Beyond hardcore gambling: understanding why mainland chinese visit casinos in macau. J. Hospitality Tourism Res. 36, 32–51. 10.1177/1096348010380600

[B96] Yu C.-E. Sun R. (2019). The role of Instagram in the UNESCO's creative city of gastronomy: a case study of Macau. Tourism Manag. 75, 257–268. 10.1016/j.tourman.2019.05.011

